# Involvement of Flagella-Driven Motility and Pili in *Pseudomonas aeruginosa* Colonization at the Air-Liquid Interface

**DOI:** 10.1264/jsme2.ME11322

**Published:** 2012-02-22

**Authors:** Kyosuke Yamamoto, Hiroyuki Arai, Masaharu Ishii, Yasuo Igarashi

**Affiliations:** 1 Department of Biotechnology, Graduate School of Agricultural and Life Sciences, The University of Tokyo, Yayoi 1–1–1, Bunkyo-ku, Tokyo 113–8657, Japan

**Keywords:** pellicle, biofilm, motility, surface adherence, *Pseudomonas aeruginosa*

## Abstract

Many aerobic microorganisms can colonize at the air-liquid interface and form a multicellular structure, known as a pellicle. In this study, the involvement of motility and attachment traits in the *Pseudomonas aeruginosa* pellicle formation process was investigated. Flagella- and flagellar-motor-deficient mutants exhibited delayed pellicle formation and unusual pellicle morphology, indicating the large contribution of flagella-driven motility to structural development of the pellicle. A pili-deficient mutant showed normal pellicle formation properties, while the disruption of the pilus gene in the flagella-deficient mutant restored normal pellicle morphology. These results indicate that flagella and pili play key roles in *P. aeruginosa* pellicle development.

The ubiquity of the sessile growth mode of microorganisms in natural environments has been widely recognized and the physiological and ecological aspects of such growth modes have attracted much recent attention. Among such sessile growth modes, the solid-liquid interfacial colonization of microorganisms, known as a biofilm, has been actively investigated in various species and communities from clinical, industrial, and ecological viewpoints (11, 15, 16). Microorganisms can settle down on not only a solid surface but also a liquid surface. Numerous aerobic microorganisms are capable of migration to air-liquid interfaces by aerotactic behavior, where they subsequently settle by forming a biofilm-like structure composed of cells and a matrix of extracellular polymeric substances, known as a pellicle (2, 14, 18). Pellicle formation is considered to be a strategy of aerobes to establish themselves within the niche at the air-liquid interface, which is selectively advantageous for aerobes due to high oxygen availability (12, 21). Although the ubiquity and significance of the microbial niche at the air-liquid interfaces in both natural and artificial environments have been pointed out (19, 20), studies on pellicle lifestyle have been relatively limited and its physiological and ecological characteristics are largely unknown.

The aim of this study was to elucidate pellicle formation mechanisms by clarifying the genetic factors which contribute to the pellicle formation process of a facultative aerobe, *Pseudomonas aeruginosa* PAO1. We especially focused on the role of motility in pellicle formation and investigated the pellicle formation properties of gene disruption mutants that impair motility-related apparatus, flagella and pili.

The bacterial strains and plasmids used in this study are listed in Table S1. *P. aeruginosa* PAO1 (WT) and its derivatives were routinely grown in LB medium at 37°C under static or shaking conditions. When required, the medium was supplemented with 100 μg mL^−1^ ampicillin for *Escherichia coli* and 300 μg mL^−1^ carbenicillin for *P. aeruginosa*. Static cultures were performed in short test tubes (Pyrex glass, inner diameter 13 mm, length 85 mm) with 3 mL medium. For the observation of pellicle morphology, a 6-well titer plate with 5 mL LB medium was used. Precultures were prepared by inoculating 3 mL medium with 1% (v/v) of an overnight culture, which was then incubated for 4 h at 37°C with vigorous shaking. Cultures were then inoculated with 1% (v/v) of preculture (optical density at 600 nm was approximately 1.4). Total pellicle mass, including the central floating portion, was quantified by a method described in the previous report (21). After gently layering 100% (v/v) methanol on the surface of a static culture, viscous pellicles were dehydrated and turned into a solid piece of rigid cell aggregation, which was then harvested using a looped wire and suspended in 200 μL phosphate buffered saline (PBS, 137 mM NaCl, 8.1 mM Na_2_HPO_4_, 2.68 mM KCl, and 1.47 mM KH_2_PO_4_, pH 7.4). After sonication for 5 min using a Bioruptor sonicator (Cosmo Bio, Japan), the total protein concentration of the suspension was determined using the bicinchoninic acid (BCA) protein assay kit (Pierce) with bovine serum albumin as a standard. Recombinant DNA experiments were performed by standard methods (13). Introduction of DNA into *P. aeruginosa* strains was carried out by transconjugation with *E. coli* strain S17-1. Ex Taq and PrimeStar polymerases (Takara, Japan) were used for polymerase chain reactions (PCRs). The primers used for PCR are listed in Table S1. Gene disruption was performed by in-frame deletion with established procedures (21). The flagellar mutant was constructed by the disruption of *fliC*, which encodes flagellin protein, as follows. The 1.1- and 1.0-kb fragments of the downstream and upstream regions of *fliC*, respectively, were amplified by PCR with the primer sets fli1-fli2 and fli3-fli4, and were then tandemly inserted into the BamHI-SalI and SalI-SphI sites, respectively, of pEX18Ap (6). The constructed plasmid, designated pEX-RMfli, was transformed into *E. coli* strain S17-1 and then transferred to PAO1 by transconjugation. A single-crossover recombinant that carried pEX-RMfli on the chromosome was selected on Pseudomonas Isolation Agar (Becton Dickinson) containing carbenicillin. A second-crossover mutant that lost the *sacB*-containing vector region was selected on an LB plate containing 5% (w/v) sucrose. After screening potential mutants by PCR analysis with primers fli1 and fli4, an in-frame *fliC* deletion mutant was identified and designated FLI1. To generate the vector construct for the disruption of *pilA*, which encodes type IV pilin protein, two 1.1-kb fragments of the upstream and downstream regions of *pilA*, respectively, were amplified by PCR with primer sets pil1-pil2 and pil3-pil4, respectively. The amplified fragments were tandemly inserted into the EcoRI-SacI and SacI-PstI sites of pEX18Ap, generating pEX-RMpil. PIL1 was constructed as above. The double mutant, FP2, was generated following the transformation of pEX-RMpil into FLI1. The flagellar motor protein mutant, MOT2, was constructed by disrupting *motAB* and *motCD* loci, which encode flagellar motor proteins, sequentially. To generate the vector construct for the disruption of *motAB*, 1.2-kb fragments of the downstream regions of *motB* and 1.1-kb fragments of the upstream regions of *motA* were amplified by PCR with primer sets mab1-mab2 and mab3-mab4, respectively. The amplified fragments were tandemly inserted into the EcoRI-KpnI and KpnI-PstI sites of pEX18Ap, generating pEX-RMmab. To generate the vector construct for the disruption of *motCD*, 1.2-kb fragments of the upstream regions of *motC* and 0.8-kb fragments of the downstream regions of *motD* were amplified by PCR with primer sets mcd1-mcd2 and mcd3-mcd4, respectively. The amplified fragments were tandemly inserted into the EcoRI-SacI and SacI-SmaI sites of pEX18Ap, generating pEX-RMmcd. Firstly, the *motCD* mutant, MCD1 was constructed with pEX-RMmcd as above. The double mutant, MOT2, was then generated following the transformation of pEX-RMmab into MCD1.

Flagella are known to be highly involved in the biofilm formation process (1, 7, 10). The contribution of flagella to pellicle formation under static conditions was evaluated by mutational analysis. A flagella-deficient strain, FLI1, was constructed by gene disruption of *fliC* and its pellicle formation properties (biomass and morphology) were investigated. FLI1 failed to form a normal pellicle that covers whole liquid surfaces and exhibited unusual pellicle morphology in the manner that cells aggregated around the solid surface (wall of culture vessel) and did not spread into the center part of the liquid surface area (Fig. 1). This result indicates the important contribution of flagella to the formation of pellicle structures on a macroscopic scale. In addition, FLI1 showed slightly delayed pellicle formation compared to WT (Fig. 2), indicating that flagella play a key role in the initial step of pellicle development.

FLI1 did not possess flagella and exhibited a defect in swimming motility (data not shown). Since flagella are cell surface appendages and involved in cell surface properties, a flagella-deficient mutant FLI1 strain may alter not only motility but also surface attachment ability or aggregability. To evaluate the contribution of swimming motility to pellicle formation, gene disruptions were performed in *mot* loci that encode flagellar motor proteins. *P. aeruginosa* possesses two gene sets for flagellar motor, *motAB* and *motCD*. As it has been reported that the products of these two gene sets have identical function and deletion of one gene set was not sufficient to diminish swimming motility (4, 17), a double mutant of these two gene loci was constructed. Flagella staining and motility assays confirmed that the obtained mutant, MOT2 strain, was a flagella-positive but swimming-negative strain (data not shown). The MOT2 strain showed pellicle formation properties comparable to those of FLI1; peculiar pellicle morphology and delayed pellicle formation (Fig. 1 and 2). This result clearly indicates that flagella-driven swimming motility is a key factor in pellicle development covering liquid surfaces. Pellicle formation has been considered to begin with attachment to the solid surface and to proceed by spreading at the air-liquid surfaces (9). After a monolayer of cells has formed, the pellicle matures and develops by cell propagation at the air-liquid interfaces and the integration of planktonic cells from the bulk phase into the pellicle. Flagella-driven motility could be involved in two migration processes, surface spreading and cell integration from the bulk liquid phase.

Since another cell surface appendage, type IV pili, is known to be involved in biofilm formation (1, 5, 7, 10), its contribution to pellicle formation was also evaluated. A pili-deficient strain PIL1 was constructed by disruption of the *pilA* gene, which was confirmed by a defect in twitching motility in soft agar (data not shown). PIL1 showed normal pellicle formation identical to that of WT (Fig. 1 and 2); however, unexpectedly, *pilA* disruption in the FLI1 strain that gave a *fliC pilA* double mutant strain, FP2, resulted in further change in the pellicle formation properties. In FP2, pellicle morphology was restored to a homogeneous sheet-like structure covering the whole liquid surface, similar to that of WT (Fig. 1). A series of morphological changes of the pellicle in motility mutants was confirmed by gene complementation analysis; *pilA* gene complementation in FP2 resulted in unusual pellicle morphology and subsequent *fliC* complementation gave a normal pellicle (data not shown). Thus, pili are also involved in the pellicle development process, while their contribution to pellicle formation could be less than that of flagella. Since pili enhance cell aggregation and surface adherence (3), they might prevent cells from spreading at the liquid surface in a swimming-motility-deficient background, resulting in the peculiar pellicle morphology observed in FLI1 and MOT2. When both flagella and pili were deficient, it was considered that cells could not move in liquid or adhere to a solid surface actively and consequently the cells at the air-liquid interfaces could be passively retained at and transferred within the air-liquid interface by physicochemical forces such as surface tension or convection. Therefore, the phenotypic changes of pellicle morphology observed in the motility mutants were considered to reflect the importance of the balance between migration and aggregation ability during the construction of pellicle structures. The final biomass of the FP2 pellicle was reduced (Fig. 2) and the pellicle seemed to be thinner than that of WT. Since the planktonic growth of FP2 was identical to that of WT (data not shown), the reduction of maximum pellicle mass in FP2 could not be caused by a simple growth defect. It is possible that EPS production was not fully induced in FP2 pellicle cells due to a decrease of the surface adhesion that stimulate cells to produce EPS to construct multicellular structures (8).

In conclusion, we have demonstrated the involvement of flagella-driven motility and pili in *P. aeruginosa* pellicle development. It has been shown that flagella and pili play key roles in *P. aeruginosa* biofilm developmental processes such as the initial attachment and the construction of a microscopic mushroom-like structure. In this study, these traits were shown to be involved in pellicle structure development at initial and maturation steps. In particular, it should be noted that flagella-driven motility governs construction of the macroscopic pellicle structure. Although the detailed mechanisms of pellicle formation remain elusive, this study revealed the significance of the physical activity of cells in unique microbial lifestyle at the air-liquid interfaces.

## Supplementary material



## Figures and Tables

**Fig. 1:**
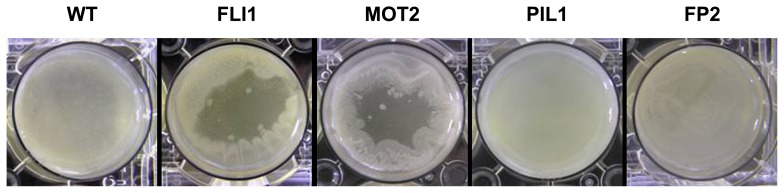
Pellicle morphology of *Pseudomonas aeruginosa* PAO1 and motility mutants. Overhead view of pellicles of WT, FLI1, MOT2, PIL1, and FP2 (left to right). Strains were statically cultivated in a 6-well titer plate with 5 mL LB medium for 22 h, except for MOT2 (40 h).

**Fig. 2:**
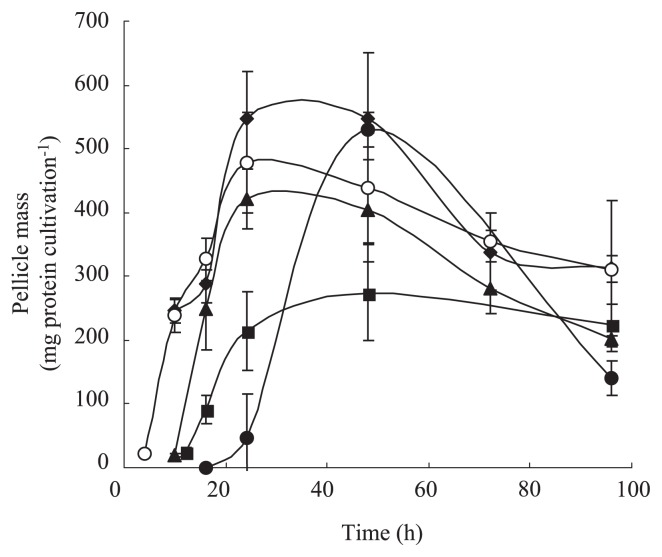
Pellicle formation by *Pseudomonas aeruginosa* PAO1 and motility mutants. WT (open circle), FLI1 (triangle), MOT2 (closed circle), PIL1 (diamond), and FP2 (square) were cultivated in LB medium under static conditions. Pellicle mass was determined by protein-based quantification. Values are expressed as means for more than three independent experiments. Error bars indicate SD.
