# Supplementary material for: Involvement of Flagella-Driven Motility and Pili in Pseudomonas aeruginosa Colonization at the Air-Liquid Interface
Source: Microbes Environ. 2012 Feb 22;27(3):320–3. doi: 10.1264/jsme2.ME11322 (PMC4036044; doi:10.1264/jsme2.ME11322)
Supplement: Supplementary file 1 [file 27_320_s1.pdf]

Supplementary material

Table S1. Strains, plasmids, and primers used in this study.

Strain, plasmid or primer	Genotype or relevant characteristic(s)	Source or reference
P. aeruginosa		
PAO1	Strain PAO1	University of Washington
FLI1	*fliC*	This work
PIL1	*pilA*	This work
FP2	*fliC pilA*	This work
MCD1	*motCD*	This work
MOT2	*motAB motCD*	This work
E. coli		
JM109	Host strain for DNA manipulation	Sambrook *et al.* (1989)
S17-1	C600::RP-4 2-(Tc::Mu)(Kan::Tn7) *thi pro hsdR hsdM*$^{+}$ *recA*	Simon *et al.* (1983)
Plasmids		
pEX18Ap	Gene replacement vector with MCS from pUC18; AP^{r}, Cb^{r}, *oriT*$^{+}$, *sacB*$^{+}$	Hoang *et al.* (1998)
pEX-RMfli	Plasmid for *fliC* mutation, a derivative of pEX18Ap	This work
pEX-RMpil	Plasmid for *pilA* mutation, a derivative of pEX18Ap	This work
pEX-RMmab	Plasmid for *motAB* mutation, a derivative of pEX18Ap	This work
pEX-RMmcd	Plasmid for *motCD* mutation, a derivative of pEX18Ap	This work
Primers		
fli1	ggc ggg atc cgc cat ggc ggc gat ctc g	This work
fli2	att ggt cga ctg ctg cag ggc acc ttc	This work
fli3	cgc tgt cga cga acc agg tgc tgc aac	This work
fli4	ttc agc atg ccg ctg ttg aac ttg gc	This work
pil1	agg cga att cca ggc caa cgt ccg cac	This work
pil2	gga tga gct ctg ggc ttg taa atc tac	This work
pil3	cga tga gct caa cca cga tca tca gtt c	This work
pil4	ggg tct gca ggg tgt tga cgc gaa agt c	This work
mab1	ccg tga att cta gcg tgg aac gcc g	This work
mab2	ctt cgg tac cgg cgg acg agg tgc ag	This work
mab3	ccg ggg tac ctg ctg aag atg ggg ctg	This work
mab4	ccc act gca gta gcc gcc agc gtt c	This work
mcd1	cga aga att ccg agg aca tct cgc gc	This work
mcd2	gat cga gct ccg cat cgg cga tgc tc	This work
mcd3	tgg tga gct cgc gca acc tgg agg tg	This work
mcd4	cgt agg tac cat ggt ata ggg caa cg	This work

Ap^{r}, ampicillin resistant; Cb^{r}, carbenicillin resistant.

Supplementary reference

Simon, R., U. Priefer, and A. Puhler. 1983. A broad host range mobilization system for in vivo genetic engineering: transposon mutagenesis in gram negative bacteria. Nat. Biotechnol. 1:784-791.
